# A markerless gene deletion and integration system for *Thermoanaerobacter ethanolicus*

**DOI:** 10.1186/s13068-016-0514-1

**Published:** 2016-05-04

**Authors:** Xiongjun Shao, Jilai Zhou, Daniel G. Olson, Lee R. Lynd

**Affiliations:** 14 Engineering Drive, Thayer School of Engineering, Dartmouth College, Hanover, NH 03755 USA; DOE BioEnergy Science Center, Oak Ridge National Laboratory, Oak Ridge, TN 37831 USA

**Keywords:** *Thermoanaerobacter ethanolicus*, Gene deletion, Gene integration, Marker removal, Clean knockout, *tdk*, FUDR, Natural competence

## Abstract

**Background:**

*Thermoanaerobacter ethanolicus* produces a considerable amount of ethanol from a range of carbohydrates and is an attractive candidate for applications in bioconversion processes. A genetic system with reusable selective markers would be useful for deleting acid production pathways as well as other genetic modifications.

**Results:**

The thymidine kinase (*tdk*) gene was deleted from *T. ethanolicus* JW200 to allow it to be used as a selectable marker, resulting in strain X20. Deletion of the *tdk* gene reduced growth rate by 20 %; however, this could be reversed by reintroducing the *tdk* gene (strain X20C). The *tdk* and high-temperature kanamycin (*htk*) markers were tested by using them to delete lactate dehydrogenase (*ldh*). During positive selection of *ldh* knockouts in strain X20 on kanamycin agar plates, six out of seven picked colonies were verified transformants. Deletion of *ldh* reduced lactic acid production by 90 %. The *tdk* and 5-fluoro-2′-deoxyuridine (FUDR) combination worked reliably as demonstrated by successful *tdk* removal in all 21 colonies tested.

**Conclusion:**

A gene deletion and integration system with reusable markers has been developed for *Thermoanaerobacter ethanolicus* JW200 with positive selection on kanamycin and negative selection on FUDR. Gene deletion was demonstrated by *ldh* gene deletion and gene integration was demonstrated by re-integration of the *tdk* gene. Transformation via a natural competence protocol could use DNA PCR products amplified directly from Gibson Assembly mixture for efficient genetic modification.

## Background

Plant biomass is the only foreseeable sustainable source of organic fuels, chemicals, and materials available to humanity [1]. Lignocellulosic biomass is particularly attractive in this context because of its widespread availability and low cost [2]. *Thermoanaerobacter ethanolicus* is a gram-positive, anaerobic thermophile which can produce a considerable amount of ethanol from a wide range of polymeric and soluble carbohydrates [3] and is of interest for bioconversion processes [4].

For commercial application of *Thermoanaerobacter* or *Thermoanaerobacterium* species, genetic engineering is likely necessary to remove by-product formation and increase the yield of final product [5–7]. Electrotransformation has been reported for *Thermoanaerobacter ethanolicus* JW200 with low transformation efficiency [8]. The discovery of natural competence and relatively high-transformation efficiency for *T. ethanolicus JW200* and many other *Thermoanaerobacter* or *Thermoanaerobacterium* species has greatly reduced the complexity of DNA manipulation in these bacteria [9]. Metabolic engineering involving multiple genetic manipulations requires either multiple selective markers, which are not described for *T. ethanolicus,* or removable selective markers. Two marker removal systems have been developed for *Thermoanaerobacterium saccharolyticum*. One is based on the *pyrF* gene conferring sensitivity to 5-fluoroorotic acid (5-FOA). The other is based on the *pta*–*ack* gene cassette conferring sensitivity to haloacetate [10]. Both of these systems have certain disadvantages. The *pyrF* system requires media without uracil, and the *pta*–*ack* system requires a pH of around 5. Therefore, neither of these systems is suitable for *T. ethanolicus*, which requires yeast extract for growth and does not grow well at pH 5.

Two additional marker removal systems have been developed in thermophilic bacterium *Clostridium thermocellum*: Hypoxanthine phosphoribosyl transferase (Hpt) encoded by *hpt* which confers sensitivity to 8-azahypoxanthine (8AZH) and thymidine kinase (Tdk) encoded by *tdk* which confers sensitivity to 5-fluoro-2′-deoxyuridine (FUDR) [11]. The *hpt* gene has also been developed as a negative selection marker for *Archaea* [12]. The *tdk* gene has a key role in the synthesis of DNA and in cell division because it is part of the pathway used to salvage thymidine from degraded DNA [13, 14]. Tdk converts FUDR to fluoro-dUMP (F-dUMP), which inhibits ThyA. ThyA catalyzes an essential reaction, converting dUMP to dTMP in nucleic acid metabolism. The *tdk* marker has been used for counter-selection in a variety of eukaryotic organisms [15–17], although its use in prokaryotes is rare (the only report to date being *C. thermocellum*). The *tdk* gene used in *C. thermocellum* is originally from *T. saccharolyticum*, indicating this strategy may also work in other thermophilic microorganisms, especially *Thermoanaerobacter* and *Thermoanaerobacterium* genus.

In this paper, we sought to develop a marker recycling system for *T. ethanolicus*, based on the positive Kanamycin selection using the *htk* marker, followed by negative FUDR selection using the *tdk* marker, which could function in the presence of yeast extract and at a neutral pH.

## Results

### Genetic basis of FUDR selection in *T. ethanolicus* JW200

Unlike *C. thermocellum*, *T. ethanolicus* has a putative *tdk* gene that encodes thymidine kinase, Teth_0051. Protein BLAST indicated that the enzyme encoded by Teth_0051 had 82 % identity to that of *T. saccharolyticum* used in *C. thermocellum* (Tsac_0324). We also found a putative gene (Teth_1237) encoding ThyA, which would be inhibited by F-dUMP.

### Deletion of *tdk* in wild-type strain

**Fig. 1:**
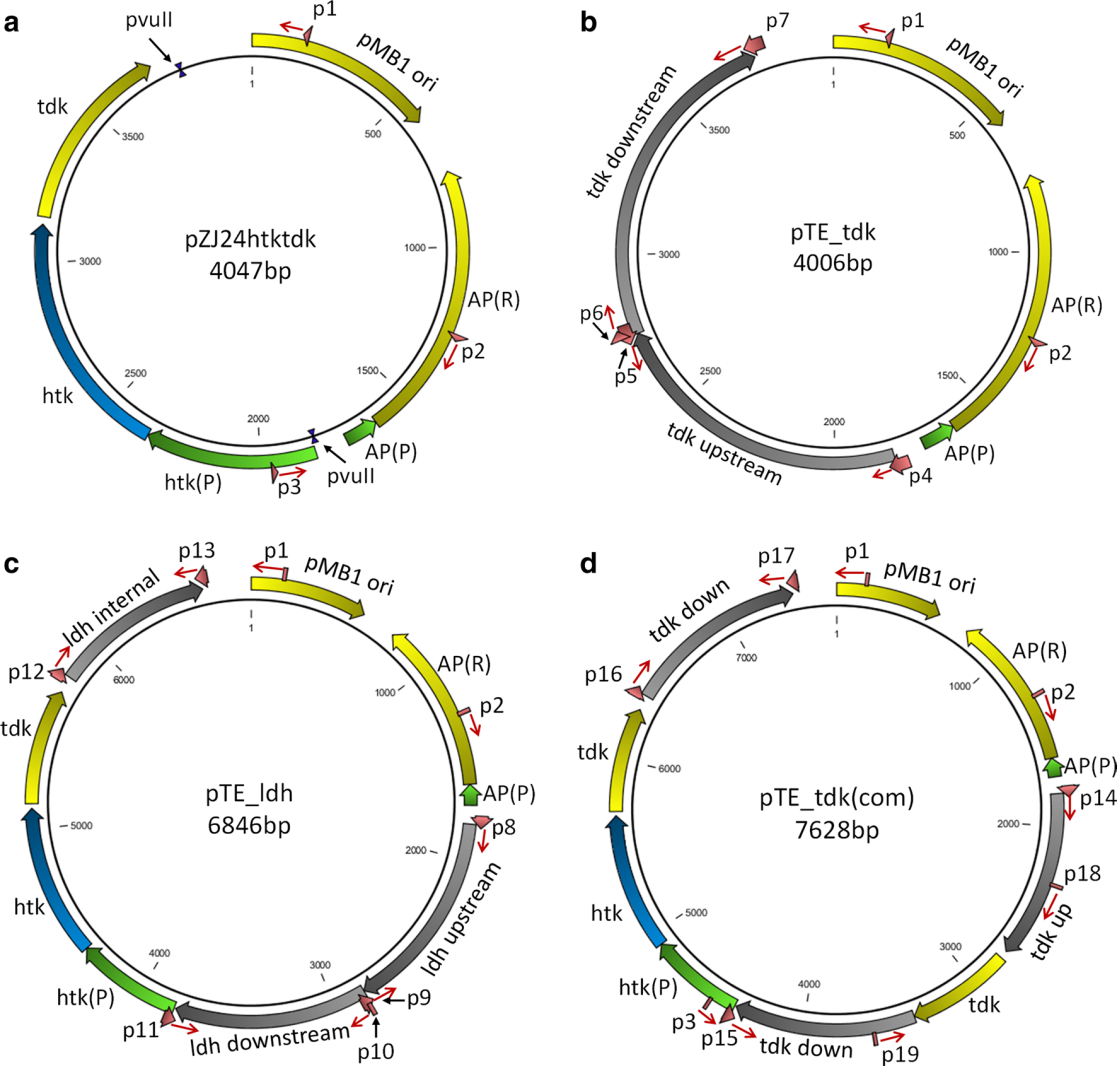
Plasmid maps for gene deletion and integration: **a** backbone plasmid, **b** plasmid for *tdk* deletion in wild-type, **c** plasmid for *ldh* deletion in stain X20, **d** plasmid for *tdk* integration in strain X20. *Red*: primers; *green*: promoters; *yellow*: coding region or origin of replication; *blue*: coding region for htk; *gray*: region for homologous recombination. *AP* ampicillin; *up* upstream; *down* downstream; *ori* origin; *R* resistance; *P* promoter; *red arrow* primer direction

**Fig. 2:**
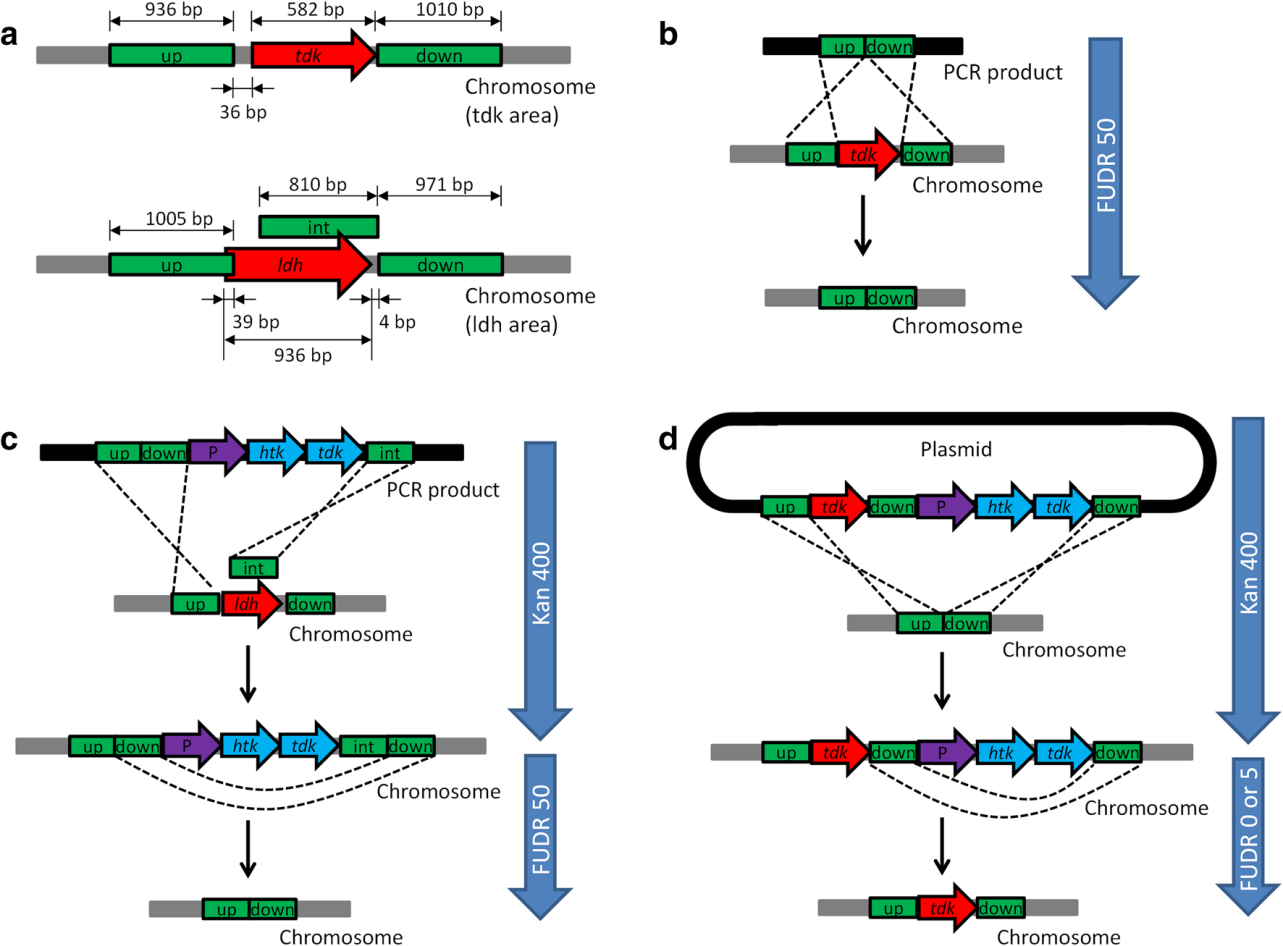
Design of homologous recombination fragments (**a**) recombination events for deleting *tdk* in wild-type strain (**b**) recombination events for deleting *ldh* in X20 (**c**) and recombination events for inserting *tdk* into X20 (**d**) *P* promoter; *up* upstream; *down* downstream; *int* internal

**Fig. 3:**
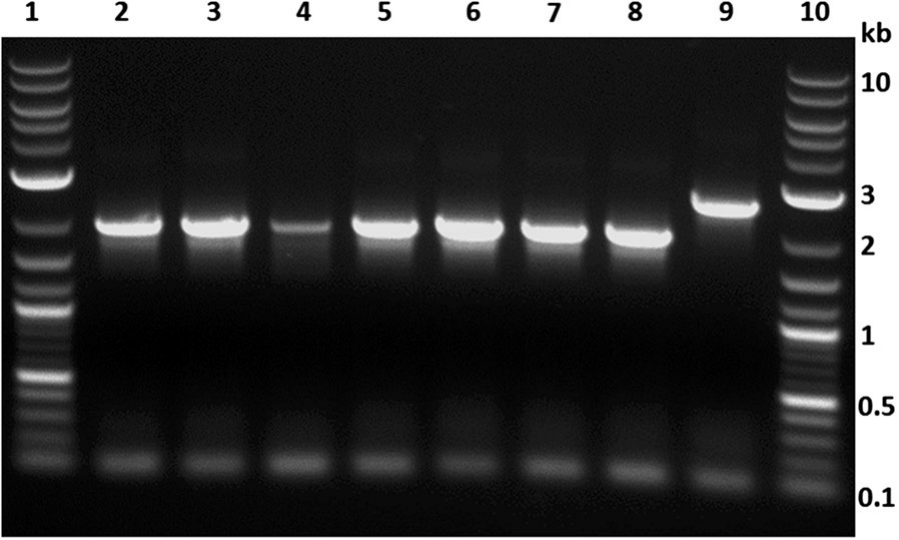
Gel confirmation for *tdk* deletion in wild-type (WT) strain. *Lanes 1 and 10*: 2-log DNA ladder; *lanes 2*–*8*: seven picked colonies; *lane 9*: WT control

To create a background strain from which an antibiotic resistance marker can be removed via negative selection, wild-type *T. ethanolicus* JW200 was transformed with PCR products from plasmid pTE_tdk (Fig. 1b) to delete the *tdk* gene. Upstream and downstream homologous recombination fragments (800–1100 bp) were selected without any overlap with the *tdk* gene and its rbs region as illustrated in Fig. 2a. Recombination events resulting in the deletion of *tdk* are shown in Fig. 2b. No colonies appeared on the agar plates with 50 mg/L FUDR when inoculated with the wild-type. Seven colonies on plates inoculated with transformed cells were picked and analyzed by colony PCR. The resulting PCR products were analyzed by DNA gel electrophoresis, which is shown in Fig. 3. All seven colonies picked from FUDR plates displayed a band size of 2.0 kb compared to a band size of 2.7 kb for the wild-type control. Confirmed by sequencing, colony 6, denoted strain X20, was chosen as the parent strain for subsequent gene deletion and integration experiments.

### Gene deletion

**Fig. 4:**
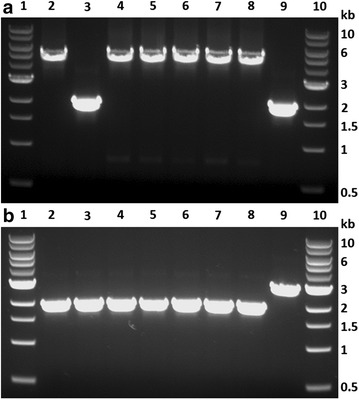
Gel confirmation for *ldh* deletion in X20. **a** *Lanes 1 and 10*: 1 kb DNA ladder; *lanes 2*–*8*: seven colonies picked from kanamycin agar plate; *lane 9*: X20 control. **b** *Lanes 1 and 10*: 1 kb DNA ladder; *lanes 2*–*8*: seven colonies picked from FUDR agar plate; *lane 9*: X20 control

To demonstrate gene deletion, the *ldh* gene was deleted in the *tdk* deletion background (strain X20). Strain X20 was transformed with PCR products from plasmid pTE_ldh (Fig. 1c). Upstream, internal, and downstream homologous recombination fragments (800–1100 bp) were selected according to primer design for PCR amplification (Fig. 2a). Recombination events resulting in the deletion of *ldh* is shown in Fig. 2c. Seven colonies were picked on CTFUD agar plates with 400 mg/L kanamycin sulfate plated with transformed culture. No colonies were observed on the kanamycin agar plate for the X20 control strain. The picked colonies were analyzed by colony PCR with the X20 strain as control. The DNA gel electrophoresis diagram loaded with colony PCR products is shown in Fig. 4a. Six out of seven picked colonies were confirmed to have the correct DNA integration, with a band size of 4.9 kb compared to a band size of 1.7 kb for the X20 control strain. At this stage, the *ldh* gene had been disrupted by the *htk*-*tdk* cassette. The next step was to remove the *htk*-*tdk* cassette by counter-selection with FUDR.

Colony 6 was chosen and plated on CTFUD agar plates with 50 mg/L FUDR to select for cells without a functional *tdk* marker. It was expected that the *tdk* and *htk* markers would be lost simultaneously. Seven colonies were picked from the FUDR agar plate. The picked colonies were analyzed by colony PCR with the X20 strain as control. The DNA gel electrophoresis diagram is shown in Fig. 4b. All seven colonies were confirmed to have the correct DNA size, with a band size of 2.0 kb compared to a band size of 2.6 kb for the X20 control strain. Colonies 2 and 7 were confirmed by sequencing and used for subsequent fermentation characterization.

### Gene integration

**Fig. 5:**
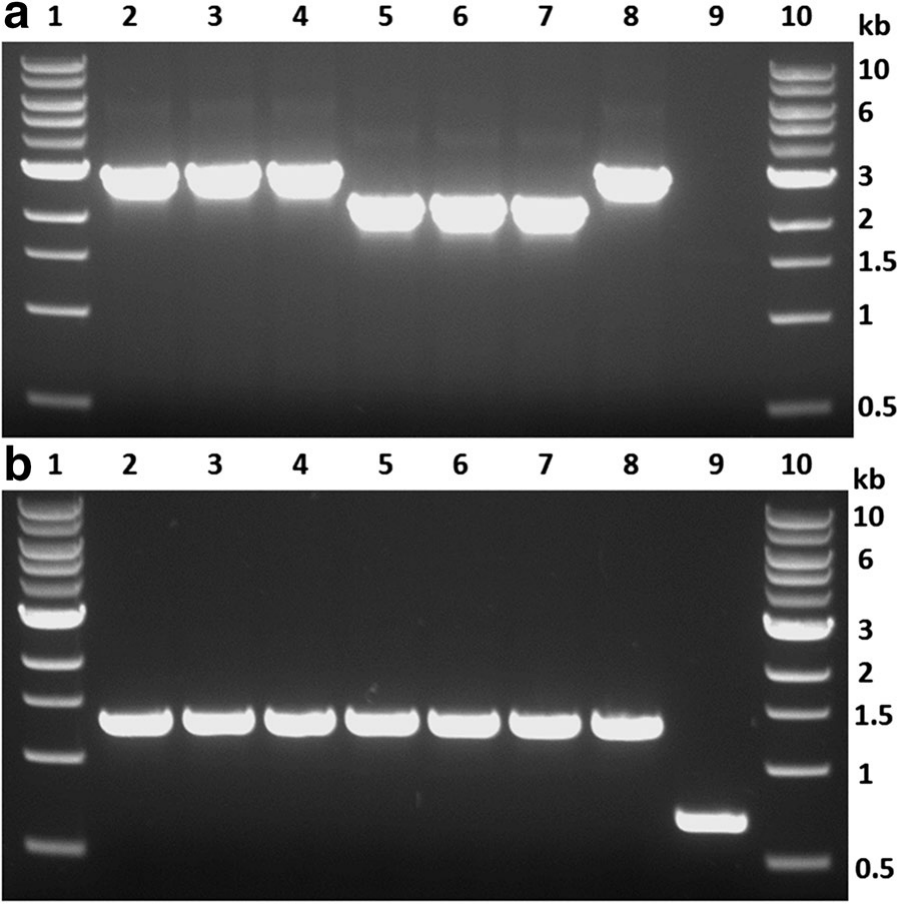
Gel confirmation for integration of *tdk* in X20. **a** *Lanes 1 and 10*: 1 kb DNA ladder; *lanes 2*–*8*: seven colonies picked from kanamycin agar plate; *lane 9*: X20 control. **b** *Lanes 1 and 10*: 1 kb DNA ladder; *lanes 2*–*8*: seven colonies picked from FUDR agar plate; *lane 9*: X20 control

To demonstrate gene integration, the *tdk* gene was inserted back into strain X20. PCR products from plasmid pTE_tdk(com) (Fig. 1d) were transformed into strain X20. Recombination events resulting in the insertion of *tdk* into strain X20 is shown in Fig. 2d. No colonies appeared on the kanamycin agar plate for the X20 control strain. Seven colonies were picked on CTFUD agar plates with 400 mg/L kanamycin sulfate. The picked colonies were analyzed by colony PCR (Fig. 5a). Four out of seven picked colonies were confirmed to have correct DNA integration, with a band size of 2.8 kb compared to the expected no band for the X20 control strain. At this point, the strain had two copies of the *tdk* marker on the chromosome: one at its native locus and one downstream of the *htk* gene.

To eliminate the second copy of the *tdk* gene, cells were plated at a low concentration of FUDR. Concentrations of 0 and 5 mg/L were tested. Seven colonies were picked from the FUDR agar plate. The picked colonies were analyzed by colony PCR (Fig. 5b). All seven colonies appeared to have correct deletions, with a band size of 1.3 kb compared to a band size of 0.7 kb for the X20 control strain. Four colonies were chosen (C1, C2, C3, and C7) for sequencing and all were confirmed to have the same sequence as the wild-type strain. Colonies 2 and 7 were selected for fermentation characterization.

### Comparison of fermentation profile

Fermentation products were measured for the engineered strains and the wild-type strain (Table 1). Two wild-type stocks were cultured, each with five replicates. Two colonies were analyzed for X20 and X32, respectively. All seven colonies picked for X20C were cultured. The two wild-type stocks had quite different fermentation product profiles, with the WT* stock having much higher organic acid production and lower ethanol yield. Both stocks were confirmed by 16S sequencing. The reason for the variation in fermentation product profile, also observed by the original researchers [3], was unknown. Compared to the WT strain, the X20 strain had about 4–5 % decrease in carbon recovery from measured fermentation products (*R*_*c*_) and a 20–25 % decrease in specific growth rate (*µ*). Deletion of *ldh* (strain X32) reduced lactate production by 90 % and increased ethanol and acetate production slightly. Compared to the X20 parent strain, there was about 4 % decrease in carbon recovery and 10 % decrease in specific growth rate. Reintroduction of the *tdk* gene in the X20 strain restored the growth rate and fermentation product profile observed in the WT strain.

**Table 1:** Fermentation profile of strains

Strain	Lactate, g/L	Acetate, g/L	Ethanol, g/L	*R*_c_	*Y*_eth_	*µ*, h^−1^
WT	0.604 ± 0.024	0.271 ± 0.029	1.655 ± 0.008	0.950 ± 0.009	0.370 ± 0.002	0.292 ± 0.009
WT^a^	1.690 ± 0.038	0.545 ± 0.028	0.880 ± 0.039	0.945 ± 0.002	0.197 ± 0.009	0.272 ± 0.011
X20	0.654 ± 0.009	0.298 ± 0.003	1.493 ± 0.000	0.900 ± 0.001	0.334 ± 0.000	0.223 ± 0.010
X32	0.068 ± 0.003	0.322 ± 0.003	1.695 ± 0.018	0.866 ± 0.010	0.379 ± 0.004	0.196 ± 0.002
X20C	0.552 ± 0.015	0.267 ± 0.009	1.649 ± 0.021	0.934 ± 0.008	0.368 ± 0.005	0.285 ± 0.003

^a^Wild-type of a different stock

## Discussion

A markerless gene deletion and integration system has been developed for *T. ethanolicus* JW200 that combines the *htk* positive selection marker and the *tdk* negative selection marker. Of 14 colonies picked from kanamycin selection plates, four were false positives. Of 21 colonies picked from FUDR selection plates, none were false positives. Four out of seven colonies were positive on kanamycin selection for *tdk* integration, while six out of seven colonies were positive on kanamycin selection for *ldh* deletion. This lower success rate for *tdk* integration was probably caused by the fact that there were two identical *tdk* downstream regions in the plasmid.

Deletion of *tdk* resulted in reduced cell growth rate (20 %) and carbon recovery (calculated from measured fermentation products). The reason why this deletion increased ethanol yield is unknown. Reintroduction of *tdk* restored cell growth rate to that of wild-type, but ethanol yield remained as that of *tdk* deletion strain. Overexpression of *tdk* is expected to be unfavorable based on the observation that homologous recombination occurred spontaneously to remove the DNA between the two downstream regions.

The discovery of natural competence in *T. ethanolicus* has greatly increased the convenience of its genetic modification. Transformation efficiency of *T. ethanolicus* was the highest among several species reported in a recent study [9]. This high-transformation efficiency makes it possible to transform with PCR products amplified directly from Gibson Assembly products, further simplifying genetic manipulation. The length of DNA fragments for homologous recombination had a large impact on the recombination frequency. We recommend using a length of 800–1000 bp based on the experience of genetic manipulations in *T. ethanolicus* and *C. thermocellum*, and further that 400–500 bp be left on both ends of the entire DNA fragment when using PCR products instead of circular plasmids. This approach, also reported by Hashimoto et al. [18], would protect the regions of homologous recombination from degradation before they reached recombination.

If the target gene for deletion is relatively small (e.g., <800 bp), an internal fragment might be too short for efficient homologous recombination. In this situation, a potential alternative gene deletion approach is to use upstream and downstream homologous recombination fragments and perform transformation twice. The first transformation uses DNA PCR products with antibiotic marker and *tdk* gene between the two upstream and downstream fragments followed by selection on agar plates with the antibiotic. Using a positive colony from the first step, the second transformation uses DNA PCR products with the two upstream and downstream fragments connected directly followed by selection on agar plates with FUDR.

The gene deletion and integration system reported here does not need manipulation of medium components and other growth conditions such as medium pH, thus greatly reducing complexity of genetic modification compared to previously described protocols [10]. It is potentially applicable to other species as long as they can express *tdk*, are sensitive to FUDR selection, have an antibiotic marker for positive selection, and are able to perform homologous recombination.

## Conclusion

A markerless gene deletion and integration system has been developed for *Thermoanaerobacter ethanolicus* JW200 with positive selection on kanamycin and negative selection on FUDR. Gene deletion was demonstrated by *ldh* gene deletion and gene integration was demonstrated by re-integration of *tdk* gene. Transformation could use DNA PCR products amplified directly from Gibson Assembly mixture for efficient genetic modification. Deletion of *ldh* gene nearly removed lactic acid production. Re-integration of *tdk* gene was desirable because its removal resulted in significant drop of specific growth rate (20 %).

## Methods

### Strains and culturing conditions

*Thermoanaerobacter ethanolicus* strain ATCC 31550 (JW200, DSM 2246) was obtained from ATCC. The strain was cultured in CTFUD medium [19] with or without 0.8 % (w/v) agar with an initial pH of 7 at 65 °C in an anaerobic chamber (Coy Laboratory Products, Grass Lake, MI).

### Construction of vectors

DNA fragments were amplified by PCR using the primers listed in Table 2. The PCR products were gel-purified. All deletion vectors used in this study were derived from plasmid pZJ24 which was created by replacing the erythromycin gene with a kanamycin gene from plasmid pZJ23 [20]. The kanamycin resistance gene in pZJ24 was replaced with a high-temperature kanamycin resistance gene (*htk*). The *htk* marker was developed by Hoseki et al. for kanamycin selection at temperatures up to 72 °C [21]. The thymidine kinase gene (*tdk*) with ribosome-binding site from *T. ethanolicus* was added downstream of the *htk* gene to generate the backbone plasmid pZJ24htktdk as shown in Fig. 1a. The backbone plasmid was digested by PvuII and column purified. The upstream, internal, and downstream regions of gene of interest for homologous recombination were amplified from *T. ethanolicus* by PCR. The PCR products and the digested backbone plasmid were assembled by Gibson Assembly. Maps for plasmids used to create the *tdk* deletion in the wild-type, lactate dehydrogenase (*ldh*) deletion in X20 strain, and *tdk* integration in X20 strain are shown in Fig. 1b–d. Complete plasmid sequences were deposited in GenBank with accession numbers KU597226, KU597227, KU597228, and KU597229.

**Table 2:** List of primers used

Name	Sequence
p1	*GAGAAAGGCGGACAGGTA*
p2	*AATTCTCTTACTGTCATGCC*
p3	*TGCCTCCTCATCCTCTTC*
p4, p14	TTAACCTATAAAAATAGGCGTATCACGAGATGCATCAG*CGCCCTGAAGAAGTAACTGACA*
p5	CCACCTATATCGGTTTTCTTCATCTCTACACCTCTTTT*AGTCTTCACCACTCTAACCCCC*
p6	*AAAAGAGGTGTAGAGATGAAGAAAACC*
p7, p17	CTCCCCGCGCGTTGGCCGATTCATTAATGATGCATCAG*GCAGTTCCGCTTCAAGTTTAGG*
p8	GACATTAACCTATAAAAATAGGCGTATCACGAGATGCATCAG*TGCCGCCTTGTACAGTTT*
p9	TTTTCACACTGTGACTTTTTATATGCAAAAAAGAGGGTTTCC*ACCGACAAATCCAGAGCC*
p10	*GGAAACCCTCTTTTTTGCAT*
p11	ATCTTACCTATCACCTCAAATGGTTCGCTGGGTTTTACGCAG*CGAACTTCCCGTAGCAAT*
p12	CTAATCTTTTCTGAAGTACATCCGCAACTGTCCATACTCCAG*TTGATATAAGCCACGGGG*
p13	CCGCGCGTTGGCCGATTCATTAATGATGCATCAGCCTCTTATATGTCAAGCTCTTGTATT
p15	TACCTATCACCTCAAATGGTTCGCTGGGTTTTACGCAGGCAGTTCCGCTTCAAGTTTAGG
p16	TCTGAAGTACATCCGCAACTGTCCATACTCCAGAAAAGAGGTGTAGAGATGAAGAAAACC
p18	*AGAAGAATTGGAGGCCAT*
p19	*CTGCAGAATAAGTAAGGCT*
p20	*TGCCGCCTTGTACAGTTT* (binding sites the same as p8)
p21	*CGAACTTCCCGTAGCAAT* (binding sites the same as p11)

Note: primer-binding regions are highlighted in italic fonts

### Gene deletion and integration

To create a background strain for negative selection, gene deletion PCR products from plasmid pTE_tdk were amplified with primers p1 and p2 from Gibson Assembly mixture, column purified, and transformed into *T. ethanolicus* wild-type strain. Colonies were picked on FUDR agar plates for colony PCR using primers p4 and p7 followed by gel confirmation.

The gene lactate dehydrogenase (*ldh*) was deleted on the *tdk* deletion background strain. PCR products from plasmid pTE_ldh were amplified with primers p1 and p2 from Gibson Assembly mixture, column purified, and transformed into *T. ethanolicus*. Colonies were picked on kanamycin agar plates for colony PCR using primers p8 and p13 followed by gel confirmation. A verified colony was chosen and plated on FUDR plates. Colonies were picked for colony PCR using primers p20 and p21 followed by gel confirmation.

The *tdk* gene was inserted back into the background strain with *tdk* deletion. Because there are two overlapping downstream regions of the *tdk* gene in plasmid PTE_tdk(com), integration PCR products could not be amplified with primers p1 and p2. Thus, Gibson Assembly mixture was transformed into DH5α competent *E. coli* (#C2987H, NEB) and plasmid was isolated for transformation. Colonies were picked on kanamycin agar plate for colony PCR using primers p14 and p3 followed by gel confirmation. A verified colony was chosen and plated on FUDR 5 mg/L or FUDR 0 mg/L plates. Colonies were picked for colony PCR using primers p18 and p19 followed by gel confirmation. Four colonies were chosen for additional confirmation by resequencing.

### Transformation and mutant selection

Transformation was performed via a natural competence protocol as described previously [9]. For positive selection of mutants with DNA integration onto genome by homologous recombination, up to 250 µl transformed culture was mixed with 20 mL CTFUD medium with 0.8 % (w/v) agar supplemented with 400 mg/L kanamycin sulfate (#420311, EMD Millipore), poured into a Falcon 100 mm petri dish (#351029, Corning), and then incubated at 65 °C after solidification. For negative selection to create the background strain X20 or to the remove antibiotic marker by homologous recombination, a confirmed colony from the positive selection step was diluted in 50 µL DI water. Up to 25 μL diluent was mixed with 5 mL CTFUD medium with 0.8 % (w/v) agar supplemented with 50 mg/L 5-fluoro-2′-deoxyuridine (FUDR) (#F0503, Sigma), poured into a 100 mm Quad-petri dish (#70686, Electron Microscopy Sciences), and then incubated at 65 °C after solidification. For each selection step, seven colonies were picked 2–3 days after incubation for colony PCR followed by gel confirmation.

### Measurement of fermentation profile

The wild-type strain together with the engineered strains X20, X32, and X20C were cultured in CTFUD medium with cellobiose at an initial concentration of 4.25 g/L in Corning culture tubes (#430157) with 5-mL reaction volume. Inoculum prepared in the same medium was added at 10 % (v/v) inoculation. The tubes were incubated at 65 °C in an anaerobic chamber for 1 day. Cell growth was measured intermittently via optical density at 600 nm with a WPA Biowave cell density meter (CO8000, Cambridge, U.K.). Samples were taken at the end for measurement of fermentation products using HPLC. Specific growth rate (*µ*) was calculated by exponential curve fitting of optical density as a function of time. Ethanol yield (*Y*_eth_) was calculated by Eq. (1) and carbon recovery (*R*_c_) was calculated by Eq. (2).

1 $$Y_{\text{eth}} = \frac{{[{\text{Ethanol}}]}}{{1.053 \times [{\text{Cellobiose}}]}}$$

2 $$R_{\text{c}} = \frac{{\left[ {\text{Lactate}} \right] + 1.5 \times \left[ {\text{Acetate}} \right] + 1.957 \times [{\text{Ethanol}}]}}{{1.053 \times [{\text{Cellobiose]}}}}$$

### Analytical methods

PCR amplification and colony PCR were both performed with Phusion High-Fidelity PCR Master Mix with HF Buffer (#M0531L, NEB). Plasmid was extracted using QIAprep Spin Miniprep Kit (#27106, Qiagen). Backbone plasmid digestion was performed with PvuII-HF (#R3151S, NEB) in CutSmart Buffer (#B7204S, NEB) at 50 °C for 15 min. Gel purification was performed on 1 % agarose gel with 0.01 % (v/v) SYBR Safe DNA Gel Stain florescent indicator (#S33102, Thermo Fisher Scientific) and recovered using Zymoclean Gel DNA Recovery Kit (#D4002, Zymo Research). Plasmids were constructed with Gibson Assembly Master Mix (#E2611L, NEB) at 50 °C for 1 h. Gels were run on Owl Easy Cast Mini Gel Electrophoresis Systems (#B1A, Thermo Scientific) for 35–40 min at 120 volts with Owl Compact Power Supply (#EC300XL, Thermo Scientific) and imaged on a Molecular Imager Gel Doc XR + System (#1708195EDU, Bio-Rad). Fermentation metabolite concentrations were determined using a Waters HPLC system equipped with an Aminex HPX-87H column (Bio-Rad Laboratories, Hercules, CA) operated at 60 °C. The mobile phase was 5 mM H_2_SO_4_ at a flow rate of 0.6 mL/min.

## Abbreviations

*tdk*: thymidine kinase gene
Tdk: thymidine kinase protein
*htk*: high-temperature kanamycin resistance gene
*ldh*: lactate dehydrogenase gene
FUDR: 5-fluoro-2′-deoxyuridine
*hpt*: hypoxanthine phosphoribosyl transferase gene
Hpt: hypoxanthine phosphoribosyl transferase protein
F-dUMP: fluoro-dUMP
ThyA: thymidylate synthase protein
WT: wild-type
